# Supplementary material for: Beyond the first glance: How human presence enhances visual entropy and promotes spatial learning
Source: PLoS Comput Biol. 2026 Jan 5;22(1):e1013173. doi: 10.1371/journal.pcbi.1013173 (PMC12799190; doi:10.1371/journal.pcbi.1013173)
Supplement: Attachment — Submitted filename: letter to the authors.pdf [file pcbi.1013173.s001.pdf]

## Summary of the paper and formulated expectations[1]

The paper presents a study conducted in virtual reality, where participants can explore a city to test differences and investigate the process of spatial cognition, specifically when learning new locations. In this VR environment, a special focus is placed on the use of social aspects, such as human-like avatars, to examine their effect on recall efficiency regarding the spatial orientation of participants. The authors claim that they are able to determine whether the presence of a social character positively influences recall accuracy in a pointing task.

In a previously published study using the same data from the same experiment, the authors had already shown that the presence of human agents and their congruency with the environment are relevant for visual exploration and knowledge acquisition (they mention this fact right-away, so transparency is provided). In the current paper, again using the same dataset, the authors aim to further explain how this contextual relevance—meaning the congruence or incongruence of their experimental manipulation—translates into changes in perception and encoding. An aspect that they left out in the previous paper, according to the authors, is the question of how congruency relates to changes in **perception and encoding (p.2, l. 25/26)**. Therefore, they intend to use vision as an indicator of how spatial information is perceived.

## Major Issues with the paper

First of all, the analysis provided in the paper appears advanced and meticulously conducted. The authors clearly understand their methods and report all relevant analytical results appropriately. The paper is also well written in terms of language and spelling, and the different analytical sections are well structured.

However, several major issues arise with this manuscript. First, it is quite unclear what significant contribution this work makes beyond the previously published article in *Frontiers in Virtual Reality*. As I understand it, the same dataset was already analyzed in the earlier paper, and the only new element here is the addition of gaze transition entropy. If this is not the case, it is particularly important that the authors explicitly state their additional contributions. At present, these are somewhat hidden behind broad terms such as "perception and encoding," "flexible gaze," or "social agent."

Another crucial aspect is that, in order to provide a meaningful contribution, the authors need to contextualize their analyses and findings more thoroughly within the discourse of (1) cognition and spatial cognition and (2) unexpected visual occurrences and (3) gaze entropy. It is worth noting that the original concept of gaze transition entropy derives from information theory, where higher entropy reflects increased unpredictability in a system (incongruent systems might be less predictable?). However, the authors do not make this connection and

[1] ChatGPT Version 4o was used to improve language and spelling in this review. Prompt used: "Improve language and spelling: "

instead focus primarily on the social aspects of the agents when interpreting incongruent and congruent differences.

Finally, I find no convincing evidence that the fact that the incongruent or concurrent objects were human-like agents played any role in the observed differences in gaze behavior. Couldn't this simply be explained by the interpretation of unexpected or unpredictable elements in the scene that naturally lead to less predictable gaze behavior? The alignment between incongruence or concurrency and the human-like nature of the objects may be coincidental rather than causal.

This, in turn, suggests that the authors' interpretation of the differences in gaze transition entropy is overly narrow in emphasizing social aspects. The argumentation could be broadened considerably, for example by considering participants' motivation, their interest, or other factors related to their performance.

Overall, I think the paper presents some interesting results regarding visual perception in virtual reality and its relation to spatial coding. However, a major revision is necessary to better contextualize the findings within the academic discourse and to ensure that the paper can be a relevant contribution to the current literature.

A more detailed evaluation of these points is provided below:

## Details Evaluation of the paper

p.2 l. 5 "Environments contain dynamic elements", Why is the human-like avatar a dynamic element? Are they moving? Especially, since the authors write in their limitation section (l. 435) "all objects and agents were non-animated."

The authors also state (p. 3, l. 55): *"Here, we investigate how the presence of human agents influences visual exploration and spatial knowledge acquisition in virtual environments."*

My interpretation of this is that the authors show that action-implying agents are associated with differences in visual exploration patterns compared to conditions without human-like agents. And, that there are differences in visual patterns between congruent and incongruent agents.

Together with the previous goal, which distinguishes this study from the already published results, these findings need to be followed up with a discussion about their implications for perception and encoding.

---

From a technical perspective, the main novelty of this paper lies in the use of gaze transition entropy. However, the theoretical contextualization of this concept and its relation to perception and encoding is not sufficiently developed.

p.2, l.38ff: The authors need to reconsider what higher gaze entropy actually indicates. Describing it as "more flexible" is not accurate, nor is referring to it simply as "more varied."

Gaze entropy is a measure used to quantify the *information content* of gaze patterns. In a scan path series, gaze entropy reflects the uncertainty or randomness (i.e., predictability) of gaze behavior. The higher the gaze entropy, the less predictable the next gaze location is.

Therefore:

- **High gaze entropy** → high randomness → a varied and wider distribution of gaze points → e.g., visual exploration.
- **Low gaze entropy** → high predictability → little variation and a focus on fewer targets → e.g., less cognitive effort or more efficient scanning (depending on the context).

Additionally, the authors do not provide a theoretical rationale for why gaze entropy should reveal insights about perception and encoding in this context. The literature review supporting this key—and possibly only—novel measurement in their study is limited.

Why did the authors not analyze stationary gaze entropy? Another indication that they lack a proper understanding of the literature on gaze entropy is that they do not even mention one of the original papers that introduced the concept in 2014 (for example, Krejtz and Duchowski, e.g., https://doi.org/10.1145/2578153.2578176). Also there have been a couple of studies since using gaze entropy as a measure in VR, e.g., https://doi.org/10.3390/s24061781, https://doi.org/10.1145/3649902.3653335, …

---

**Method:**

p. 5, l. 146 While the text describes "eight visual categories:", in Figure 2B there are only 3 categories shown?

---

**Results:**

It is hard to discern the difference between the overlapping results presented in the previous papers and this paper by the same authors:

Previous paper (in *Frontiers in Virtual Reality*):
"Furthermore, our analysis revealed that the **longest gaze times were directed toward contextual agents that mismatched their surroundings**. … When these agents clashed with their background, it likely elicited an expectancy violation (Burgoon, 2015), prompting participants to engage in a longer visual search to reconcile this disparity. … Together, these results support the notion that agents are perceived contextually, and participants showed signs of trying to integrate them into the space they inhabit. These findings align with the Dwell Hypothesis, confirming that **participants allocated more visual attention to elements with higher contextual relevance**."

This paper:
"The results revealed that agents significantly shaped visual exploration patterns, especially when incongruent with their surroundings. **Incongruent agents attracted longer dwell times and increased gaze transitional entropy**, promoting more dispersed and less predictable exploration patterns. This suggests that contextual mismatches between agents **and their environment trigger attentional shifts beyond the immediate fixation**, leading to more distributed scanning behavior."

---

Main concern for **Time analysis of GTE: Agent effects on entropy**

GTE as a measure increases when more objects of interest are visited. The authors claim to perform a temporal event separation, but what they are actually doing is an object separation. Naturally, gaze transition entropy will be higher when an additional object is fixated compared to before, simply by how the measure is calculated.

*When the interval separation is essentially just the number of objects that have been fixated on, GTE becomes confounded and is predictably higher as soon as one more object is included?*

Maybe the agent is the starting point of a visual exploration of the scene? How is this evidence for the importance of social agent?

The same issue applies to the congruent condition (p 9, l. 284ff). There were only congruent objects of interest, which means one category is missing. Additionally, the entropy values in Figure 4 for the eight different categories do not present a clear pattern. To me, they seem to show a very similar distribution, despite the different objects of interest.

P.13 l. 379 "Together, these findings reveal that agent congruency significantly modulates post-fixation gaze behavior". How can the authors know that this is significant from a Bayesian analysis?

---

Main concerns for **Performance prediction using entropy and dwell times**

**Line 413:**
"In summary, these results highlight gaze entropy as a robust predictor of spatial recall accuracy."

Why couldn't higher gaze transition entropy also reflect greater interest in exploring the scene, taking the task more seriously, or being more motivated? The conclusion is formulated in quite a one-dimensional way.

---

**Discussion and Conclusion**

P 16 l. 526 “In conclusion, spatial cognition is not solely driven by geometry or task demands but is fundamentally shaped by the social meanings embedded in our surroundings.” How is this question answered?

It is unclear why the social aspect is considered important for spatial learning in this context. Couldn’t the effect simply be due to the presence of something incongruent? The authors would only know this by manipulation something non-social as congruent or incongruent and compare the results.

From a logical perspective, the statement “Noses improve spatial cognition” is just as valid as saying “Social agents improve spatial cognition,” simply because both are present in the environment.

l. 476: I fully agree with the authors’ perspective on exploration versus exploitation, unexpected events, and the eye movement patterns these events can elicit. However, it is not clear to me how this can be attributed specifically to the fact that the agents are social. There is no evidence showing that the same effect would not occur if incongruent puppies or other objects were used in the scene. In my opinion, the authors should rephrase all relevant paragraphs to avoid the logical fallacy of assuming that the social nature of the agents is causing these effects, as they do not provide evidence supporting this claim?

L. 511: “These findings contribute to the growing discussion on the role of humans as environmental facilitators during spatial learning”, Unfortunately, I don’t think so!
