# Supplementary material for: Beyond the first glance: How human presence enhances visual entropy and promotes spatial learning
Source: PLoS Comput Biol. 2026 Jan 5;22(1):e1013173. doi: 10.1371/journal.pcbi.1013173 (PMC12799190; doi:10.1371/journal.pcbi.1013173)
Supplement: Attachment — Submitted filename: P2P entropy paper.pdf [file pcbi.1013173.s002.pdf]

Dear Editor,

Please find enclosed our revised manuscript, “Beyond the First Glance: How Human Presence Enhances Visual Entropy and Promotes Spatial Learning,” for consideration at *PLOS Computational Biology*. We are grateful for the thoughtful and constructive feedback from you and the reviewers, which has substantially improved the clarity and contribution of our work. In revising the manuscript, we addressed all issues raised. Specifically, we incorporated a counterfactual mediation analysis to examine the proposed mechanism, clarified the theoretical framing, provided the full model specifications, standardized iterations across models to ensure comparability, expanded the discussion of gaze transition entropy and its foundations, and strengthened transparency by improving our data and code documentation. We believe these revisions substantially improved the manuscript and further reinforced the robustness and relevance of our findings.

A detailed point-by-point response accompanies this submission. We are providing a revised manuscript and a tracked-changes version for ease of review. All authors have approved the resubmission and declare no competing interests. Data and code supporting the results are available at the repository indicated in the manuscript.

Thank you for your consideration. We appreciate the opportunity to revise our work and hope the updated version meets the journal’s standards and resolves any remaining concerns.

Sincerely,

Tracy Sánchez Pacheco (corresponding author)
Institute of Cognitive Science, University of Osnabrück

Lines correspond to the revised manuscript with tracked changes

Reviewer #1 Comment 1:

> *"My one bigger issue concerns the untangling of the effects of incongruency's cognitive effects, and of increased gaze entropy as its behavioural consequence. The authors demonstrate that participants were better at remembering locations of incongruent agents over congruent or acontextual ones. As the hierarchical models show, both agent incongruence and GTE are predictive of pointing accuracy towards remembered locations, with GTE being clearly the strongest predictor. However, the authors then write: 'This suggests that incongruent agents—despite violating expectations—may enhance memory through increased gaze dispersion or salience.' (l.411) There are several issues with this. (i) There is literature to suggest that incongruence may help memory under certain conditions (e.g., O'Sullivan & Durso, 1984). But more importantly, (ii) this suggests that incongruence affects memory performance only or at least primarily through altered gaze behaviour, but should the modelling not provide independent contributions of each predictor? Note that I am also missing more explicit formulations of the models in the paper, so perhaps these could help clarify. In any case, if the models do not provide this, then this would complicate the interpretation of other parts of the analyses, as dwell-time measures would also be correlated with other predictors. Point (ii) is hinted at in several other places including the abstract but, unless I am missing or misunderstanding something (which is entirely possible), not really supported by the analyses. I would ask the authors to clarify, and be precise in their interpretations."*

Done: Thank you for this thoughtful comment. We agree and have: (i) made all models explicit, presenting formulas that state contrast coding and the full random-effects structure **(p.7, Eq.5; p.8, Eqs.6-7)**; (ii) clarified independent (conditional) contributions in the primary outcome model by including agent type and GTE simultaneously (with z-scored dwell covariates) and revising the prose to avoid causal overstatement **(p.9, Eq. 8)**; and (iii) added a single-mediator counterfactual mediation (GTE) reporting NIE, NDE, TE, and corresponding ratios, with counterfactuals averaged over context and dwell covariates **(p.9, l.281–p.11, l.373)**.

Reviewer #1 Comment 2:

> *"I commend the authors for making their data and code available. However, while much of the raw data is relatively straightforward, the*

data would benefit from some description, especially given that there is not the same number of data files per participant as there are experimental blocks. It is also not clear to me why the code is stored separately."

Done: We appreciate the reviewer's positive feedback and their suggestion to improve transparency. We have now added a detailed README file to the repository in the wiki section, which explains the file structure, naming conventions, and the relationship between participants, sessions, and the number of files. This includes a clarification that, in some cases, session files were split due to technical restarts or anonymization. We have also clarified in the README that the code is stored in a separate directory for versioning (GitHub).

Reviewer #1 Comment 3:

"l.136: Please describe the algorithm here; this is not something the reader should have to go to a different paper for."

Done: Thank you for bringing this to our attention. We now include an explicit description of how the velocity-based algorithm classifies the eye-tracking data on **p.5, l.145–150.**

Reviewer #1 Comment 4:

"Are the SDs for fixation duration across agents or across participants?"

Done: Thank you for the request for clarification. The reported means and standard deviations are computed across participants within each agent condition. We have made this explicit in the text on **p.12, l.397-400**.

Reviewer #1 Comment 5:

Why are acontextual agents not mentioned at all in ll.285-304?

Done**: We have now included a summary of the acontextual agents on **p.14, l. 447-449.

Reviewer #1 Comment 6:

Please explicitly specify the full hierarchical models.

Done**. We have now expanded the explicit report of the models on **p.7, l.219-p.8, l.232; p.8, l.239-253; p.8, l.258- p.9, l.267; p.9, l. 268-280.

Reviewer #1 Comment 7:

"l.203: State the default priors, please."

Done:. We now explicitly state the priors **(p.7, l.209-214)**.

Reviewer #1 Comment 8:

"eq. 5, eq. 6: In the context of hierarchical modelling, using sigma is potentially confusing here."

Done: We agree that clarity was needed. We have revised the notation to avoid overloading σ: the **s**ession-level random intercept is now written as $v_{p:s}$ (variance σ_v^2), reserving σ for variance/scale parameters. This update is reflected on **p. 7 (Eq. 5) and p. 8 (Eq. 6)**.

Reviewer #1 Comment 9:

"Conditions are sometimes capitalised (e.g., l.227, figure caption for fig. 3), other times not (e.g., l.212, most instances in the main text). Please be consistent."

Done: Thank you for pointing out this inconsistency. We have standardized the capitalization of all condition labels (now written in lowercase, except when appearing at the beginning of a sentence).

Reviewer #1 Comment 10:

"Fig. 3: It is almost impossible to tell in the resolution provided what object the agent is holding (knowing that Fig. 3b is congruent, I assume it is a sandwich?), which appears to be central information. Larger panels A-C could help, but also a more informative figure caption."

Done: We appreciate the reviewer's attention to visual clarity. The Panels A and B now show only the agent, and the caption now includes that the object is a sandwich and specifies that B.1 is context congruent and B.2 is context incongruent has been updated **(p.13, Fig. 3)**.

Reviewer #2 Comment 1:

"First, it is quite unclear what significant contribution this work makes beyond the previously published article in Frontiers in Virtual Reality. As

I understand it, the same dataset was already analyzed in the earlier paper, and the only new element here is the addition of gaze transition entropy. If this is not the case, it is particularly important that the authors explicitly state their additional contributions. At present, these are somewhat hidden behind broad terms such as "perception and encoding," "flexible gaze," or "social agent.".

Done. We acknowledge the reviewer's concern regarding the novelty relative to our *Frontiers in Virtual Reality* article. Although both studies draw on the same dataset, the present manuscript provides substantial conceptual and methodological extensions beyond the original scope. We now state them explicitly on **p.3, l. 72–76**.

Reviewer #2 Comment 2:

"Another crucial aspect is that, in order to provide a meaningful contribution, the authors need to contextualize their analyses and findings more thoroughly within the discourse of (1) cognition and spatial cognition and (2) unexpected visual occurrences, and (3) gaze entropy. It is worth noting that the original concept of gaze transition entropy derives from information theory, where higher entropy reflects increased unpredictability in a system (incongruent systems might be less predictable?). However, the authors do not make this connection and instead focus primarily on the social aspects of the agents when interpreting incongruent and congruent differences.

Finally, I find no convincing evidence that the fact that the incongruent or concurrent objects were human-like agents played any role in the observed differences in gaze behavior. Couldn't this simply be explained by the interpretation of unexpected or unpredictable elements in the scene that naturally lead to less predictable gaze behavior?"

Done. We agree that our effects need not depend uniquely on the agents' social nature. We therefore clarify that humanness is not a necessary condition for the observed visual reorganization, but our data shows that it is a sufficient manipulation that reliably induces contextual anomaly. To address these points, we (1) explicitly anchor GTE in information theory, treating higher GTE as increased unpredictability in fixation transitions **(p.2, l. 36-53)**; (2) broaden the literature review to include more visual anomalies/semantic violations research **(p.20, l. 646-653)**; and (3) articulate alternative mechanisms that could produce similar patterns without requiring social content **(p.20, l. 665-675**; **p.21, l. 686-688)**.

Reviewer #2 Comment 3:

"p.2 l.5 "Environments contain dynamic elements", Why is the human-like avatar a dynamic element? Are they moving? Especially, since the authors write in their limitation section (l. 435) "all objects and agents were non-animated.""

Done: We thank the reviewer for pointing out this potential ambiguity. To avoid misinterpretation, we revised the sentence on **p.2, l. 5-6** to clarify that "dynamic" refers to semantic/contextual variability rather than physical movement. Here, it means that our real-life experience tells us that humans are mobile, limiting their use as potential landmarks. The term "dynamic" is used in this semantic sense to differentiate elements that are permanent in the landscape, like buildings, from elements that have the potential to change (i.e., dynamic).

Reviewer #2 Comment 4:

"The authors also state (p. 3, l. 55): "Here, we investigate how the presence of human agents influences visual exploration and spatial knowledge acquisition in virtual environments." [...] these findings need to be followed up with a discussion about their implications for perception and encoding."

Done: We expanded both the Introduction and Discussion to interpret the results in the light of navigation as policy selection balancing exploration and exploitation **(p.2, l. 36-53; p.20, l.629-634**). We refrain from overstating perception and encoding to avoid speculation.

Reviewer #2 Comment 5:

"From a technical perspective, the main novelty of this paper lies in the use of gaze transition entropy. However, the theoretical contextualization of this concept and its relation to perception and encoding is not sufficiently developed. p.2, l.38ff: The authors need to reconsider what higher gaze entropy actually indicates. Describing it as "more flexible" is not accurate, nor is referring to it simply as "more varied."

Done: We have revised the introduction to declare our interpretation of gaze transition entropy (GTE) explicitly in its mathematical definition. We now explain that GTE is computed from fixation–transition matrices and quantifies the conditional uncertainty of the next fixation given the current one **(p.2, l. 38-40)**, reaching its maximum when transitions are equally distributed across all categories **(p.2, l. 43-45)**. We also clarify how this information-theoretic property links to policy selection (**p.2, l. 47-53**). After this formal introduction, we use terms such as "flexibility" or "variety" as an interpretive/reader-friendly way to convey the consequences of this formal structure.

Reviewer #2 Comment 6:

> *"Why did the authors not analyze stationary gaze entropy? Another indication that they lack a proper understanding of the literature on gaze entropy is that they do not even mention one of the original papers that introduced the concept in 2014 (for example, Krejtz and Duchowski, e.g., https://doi.org/10.1145/2578153.2578176). Also there have been a couple of studies since using gaze entropy as a measure in VR, e.g., https://doi.org/10.3390/s24061781, https://doi.org/10.1145/3649902.3653335, ... "*

Addressed: We acknowledge the reviewer's concern for what he interprets to be a lack of proper understanding of the literature. Our choice to analyze gaze transition entropy (GTE) rather than stationary gaze entropy (SGE) was deliberate and tied to our question about dynamics immediately following an agent encounter. GTE captures how unpredictable the sequence of gaze shifts becomes, which is precisely what we seek to test when agent placements violate expectations. By contrast, SGE summarizes long-run occupancy dispersion across AOIs and is less sensitive to short, context-triggered policy changes in the immediate post-encounter interval. We now make this rationale explicit in the Introduction **(p.2, l.36–46)**. We have also added the foundational citation to Krejtz et al. on **(p. 2, l.41).**

Reviewer #2 Comment 7:

> *"p. 5, l. 146 While the text describes "eight visual categories:", in Figure 2B there are only 3 categories shown?"*

Done: Thank you for pointing us to this possible source of confusion. Figure 2B presents a simplified subset for illustration. We revised the caption and legend to state this explicitly **(p.6, Fig. 2)**.

Reviewer #2 Comment 8:

> *It is hard to discern the difference between the overlapping results presented in the previous papers and this paper by the same authors: "*
>
> *Previous paper (in Frontiers in Virtual Reality): "Furthermore, our analysis revealed that the longest gaze times were directed toward contextual agents that mismatched their surroundings. ... When these agents clashed with their background, it likely elicited an expectancy violation (Burgoon, 2015), prompting participants to engage in a longer visual search to reconcile this disparity. ... Together, these results support the notion that agents are perceived contextually, and participants*

showed signs of trying to integrate them into the space they inhabit. These findings align with the Dwell Hypothesis, confirming that participants allocated more visual attention to elements with higher contextual relevance.

This paper: "The results revealed that agents significantly shaped visual exploration patterns, especially when incongruent with their surroundings. Incongruent agents attracted longer dwell times and increased gaze transitional entropy, promoting more dispersed and less predictable exploration patterns. This suggests that contextual mismatches between agents and their environment trigger attentional shifts beyond the immediate fixation, leading to more distributed scanning behavior."

Addressed: The present manuscript provides substantial conceptual and methodological extensions beyond the original scope, aiming at a different scientific question. We now state them explicitly on **p.3, l. 72–76**.

Reviewer #2 Comment 9:

"Main concern for Time analysis of GTE: GTE as a measure increases when more objects of interest are visited. [...] How is this evidence for the importance of social agent?"

Addressed: We agree that, in principle, gaze transition entropy (GTE) would rise if participants simply encountered more objects of interest. In our study, however, this potential confound is controlled by design. Both conditions used the same virtual city with the same number of total objects, the same number of target buildings, the same number of agents, and the same exploration duration. The only difference between conditions was the spatial placement of contextual agents (congruent vs. incongruent shuffling). We have emphasized this in the manuscript on **(p.4, l.129- 131).**

Reviewer #2 Comment 10:

"P.13 l.379: How can the authors know that this is significant from a Bayesian analysis?"

Done: We used "significant" to denote that the posterior excludes zero and have now revised the wording on **p.12, l.403; p.12, l.420; p.16, l. 503; p.16, l. 507; p.16, l. 517; p.17, l. 533; p.17, l. 543-545.**

Reviewer #2 Comment 11:

> *"Line 413: Why couldn't higher gaze transition entropy also reflect greater interest in exploring the scene, taking the task more seriously, or being more motivated? The conclusion is formulated in quite a one-dimensional way."*

Done: We agree these are plausible interpretations and have now included them in the discussion **(p.20, l. 665-6675)** and conclusion **(p.21, l. 686-688)**.

Reviewer #2 Comment 12:

> *"l.476: I fully agree with the authors' perspective on exploration versus exploitation, unexpected events, and the eye movement patterns these events can elicit. However, it is not clear to me how this can be attributed specifically to the fact that the agents are social. [...]"*

Done: We thank the reviewer and agree that our results do not isolate sociality. We now explicitly state that social features were held constant, and differences in gaze patterns arise from contextual mismatch **(p.21, l. 669-671)**.
