# Supplementary material for: Beyond the first glance: How human presence enhances visual entropy and promotes spatial learning
Source: PLoS Comput Biol. 2026 Jan 5;22(1):e1013173. doi: 10.1371/journal.pcbi.1013173 (PMC12799190; doi:10.1371/journal.pcbi.1013173)
Supplement: Attachment — Submitted filename: P2P_minor_revision_entropy.pdf [file pcbi.1013173.s003.pdf]

Dear Editor,

Please find enclosed our revised manuscript, *"Beyond the First Glance: How Human Presence Enhances Visual Entropy and Promotes Spatial Learning,"* submitted for further consideration at PLOS Computational Biology. We are grateful for the positive and constructive feedback provided by you and the reviewers.

In this revision, we have addressed the issue raised and clarified the phrasing regarding the implications of our previous findings. These changes improve the precision and accuracy of the manuscript's theoretical framing.

A detailed point-by-point response to the reviewer comment accompanies this resubmission. We also provide both a clean, revised manuscript and a tracked-changes version to facilitate review. All authors have approved the revised submission and declare no competing interests. Data and code supporting the results remain available at the repository cited in the manuscript.

Thank you for considering our revised work. We appreciate the opportunity to improve the manuscript and hope that the revisions satisfactorily address all remaining concerns.

Sincerely,

Tracy Sánchez Pacheco (corresponding author)
Institute of Cognitive Science, University of Osnabrück

Reviewer #2 – Comment 1.

Reviewer comment:

> *Lines 14–16: I would appreciate more clarification here. If I understand correctly, the sentence suggests that the presence of a human agent (as opposed to the absence of an agent) influences exploration, visual attention, and memory recall, according to Sánchez-Pacheco et al. (2025). However, in Sánchez-Pacheco et al. (2025), only exploration was compared between agent vs. no-agent conditions; visual attention and memory recall were examined only between different types of agents, not presence vs. absence. Therefore, I am not sure the authors can make this claim.*

Done: We thank the reviewer for this clarification. We have revised the paragraph to more accurately reflect the findings of Sánchez-Pacheco et al. (2025). The updated text now specifies that agent presence was associated only with local differences in exploration (see Sánchez-Pacheco et al. (2025), Section 2.1.3 *Agent-induced bias on walking strategies*), whereas the semantic relevance and contextual congruence of the agents influenced visual attention and recall outcomes. We now emphasize that the agent-type (i.e., semantic relevance) effect is the main source of results of our previous work explicitly in both the introduction **(p. 2, line 12, lines 14–18; p. 2, lines 26–28)** and the discussion **(p. 20, lines 617–618; p. 20, lines 632–633)**.
